# Preoperative Health Status and Clinical Predictors of Health-Related Quality of Life Improvement After Lumbar Spinal Stenosis Surgery: A Longitudinal Study

**DOI:** 10.3390/jcm14134391

**Published:** 2025-06-20

**Authors:** Irene Ciancarelli, Alex Martino Cinnera, Alessandro Ricci, Marco Iosa, Antonio Cerasa, Rocco Salvatore Calabrò, Giovanni Morone

**Affiliations:** 1 Department of Life, Health and Environmental Sciences, University of L’Aquila, 67100 L’Aquila, Italy; irene.ciancarelli@univaq.it (I.C.); giovanni.morone@univaq.it (G.M.); 2 IRCCS Santa Lucia Foundation Hospital, 00179 Rome, Italy; marco.iosa@uniroma1.it; 3 Department of Neurosurgery, San Salvatore Hospital, ASL Avezzano-Sulmona-L’Aquila, 67100 L’Aquila, Italy; aricci@asl1abruzzo.it; 4 Department of Psychology, Sapienza University of Rome, 00185 Rome, Italy; 5 Department of Biomedical Sciences, National Research Council (CNR), 00187 Rome, Italy; antonio.cerasa@cnr.it; 6 Institute of BioImaging and Complex Biological Systems (IBSBC-CNR), 88100 Catanzaro, Italy; 7 S. Anna Institute, 88900 Crotone, Italy; 8 IRCCS Centro Neurolesi “Bonino Pulejo”, 98124 Messina, Italy; roccos.calabro@irccsme.it

**Keywords:** quality of life, health status, lumbar spinal stenosis, spinal diseases, spinal surgery, patient outcome assessment

## Abstract

**Background/Objectives:** Limited research has examined the relationship between preoperative health status and health-related quality of life (HRQoL) in patients with lumbar spinal stenosis (LSS) undergoing surgery. This study aims to assess the role of clinical, preoperative health and demographic factors on short-term HRQoL and functional outcomes following LSS surgery. **Methods**: A longitudinal study was conducted on 61 LSS patients (mean age 72.2 ± 8.8 years) undergoing surgery, assessing HRQoL and clinical outcomes before and 30 days post-surgery. Demographic and preoperative health status data were collected at baseline. HRQoL was measured using the Short Form Health Survey 36 (SF-36); clinical evaluations included assessments of disability, pain, and psychological status. Changes in HRQoL and clinical scores were analyzed with repeated measures ANOVA. HRQoL improvement was correlated with demographic and clinical variables, using Pearson’s correlation. **Results**: Spinal surgery for LSS led to significant improvements in HRQoL, with notable gains in both physical and mental health components (both *p* < 0.001), and in particular, in the body pain (+34%) and physical functioning, role physical, and social functioning (+20%) subscales of SF-36. Clinical scores also showed significant post-surgery improvements, strongly correlating with HRQoL. Correlations between ΔSF-36 subscale scores and preoperative factors revealed negative associations with BMI, smoking, comorbidities, and psychological distress. Conversely, physical activity was positively correlated with HRQoL improvements, especially in items showing the greatest score increases. **Conclusions**: Surgical treatment for LSS determines a significant improvement in HRQoL and functional outcome, which are however influenced by preoperative factors such as psychological distress, high BMI, smoking, and comorbidities. Conversely, regular physical activity is associated with better daily functioning, work performance, and social engagement. A comprehensive preoperative assessment may be a useful and appropriate tool to identify patients who are most likely to benefit and optimize quality of life after LSS surgery.

## 1. Introduction

Lumbar spinal stenosis (LSS) is defined as a syndrome of narrowing of the spinal canal, lateral recess, or neural foramina, affecting up to 47% of individuals over 60 years of age [1], and further challenged by a significant socio-health issue due to its substantial impact on quality of life [2]. In the LSS, bone, ligaments, and synovial elements of the lumbar axial spine degenerate and overgrow, progressively compressing the vascular and neural structures in the spinal canal. This narrowing can be due to several factors, including degenerative processes, such as those related to aging or overuse due to inappropriate mechanical loads, or congenital conditions [3]. If the stenosis is clinically relevant, it may cause lumbar pain, lumbar radiculopathy (with pain and sensory motor deficits), and neurogenic claudication with the compression of the nerve roots of the cauda equina [4]. In this case, patients typically experience and report activity-related lower back and leg pain that worsens with prolonged standing or walking, thus limiting walking distance and global motor performances. For these reasons, LSS is associated with a strong impairment in various areas of quality of life, particularly in ability to carry out work and daily life activities. The reduction in quality of life may be accompanied by depression and anxiety, which result in dysfunctional coping long-term strategies and psychopathological symptoms, compromising general functioning [5].

When conservative treatment fails to improve the patient’s symptoms, surgical procedures with decompression, laminectomy, and minimally invasive approaches are recommended even in older subjects [6]. However, the outcomes of these surgeries’ procedures on the improvement of specific symptoms are influenced by multiple factors, including preoperative general health and psychological status, and in particular, depression and anxiety [7], which are highly prevalent among individuals with chronic pain, with a biunivocal interaction. Preoperative depression was associated with severity of some symptoms as pain, weakness, balance impairment, and thus heavy disability [8]. Despite these assumptions, limited research has explored the specific relationship between preoperative general health and mental status and their impact on health-related quality of life (HRQoL) in LSS patients undergoing surgery. In fact, although prognostic factors for walking capacity in subjects that undertake LSS surgery has been extensively explored, few researchers have tried to identify prognostic factors for the subject’s HRQoL [9]. HRQoL is a subjective, multidimensional concept reflecting how individuals perceive the impact of their health on physical, mental, and social well-being. As a self-reported measure, it offers a patient-centered evaluation of health outcomes, providing insight into the real-world effects of medical or surgical interventions [10]. This makes HRQoL valuable for guiding clinical decisions, personalizing treatment, and informing healthcare providers about the broader effectiveness of care. Due to these characteristics, HRQoL has increasingly gained attention in recent years as a critical variable in both clinical research and practice.

Despite the high prevalence of LSS in the adult-elderly population, the assessment of the quality of life in these subjects after and post-surgery is poorly documented in the literature and rarely assessed in the clinic. In support of this assumption, it has been verified that also objective MRI findings and radiographic spinopelvic parameters are poorly correlated to quality of life, thus confirming that treatment recommendations for symptomatic LSS should never be based on radiological data only [11].

Instead, an evaluation of specific preoperative clinical and functional factors could constitute an important tool for neurosurgeons to identify the profile of LSS patients, more susceptible to a postoperative functional improvement, also in terms of HRQoL. Indeed, surgical treatment of LSS has been shown to determine a significant improvement in HRQoL and functional outcome, always in relation to preoperative conditions such as psychological distress, high BMI, smoking, and comorbidities. Conversely, regular physical activity is associated with better daily functioning, work performance, and social engagement. Therefore, a comprehensive preoperative assessment may be a useful and appropriate tool to identify patients who are most likely to benefit and optimize quality of life after lumbar stenosis surgery.

The aim of the present study is to assess the preoperative health status and identify clinical predictors of improvement in health-related quality of life following LLS surgery, with the ultimate goal of optimizing spinal surgical outcomes.

## 2. Materials and Methods

Patients with LSS candidates for spinal surgery were evaluated longitudinally at the Neurosurgery Department of the San Salvatore Hospital of L’Aquila. Inclusion criteria were patients aged 18 years or older with a diagnosis of LSS attributable to degenerative lumbar spine conditions, such as degenerative disc disease, osteoarthritis with associated osteophyte formation, or congenital spinal canal narrowing. Exclusion criteria were patients younger than 18 years; LSS resulting from traumatic or neoplastic spinal pathology; and patients with a documented diagnosis of active rheumatologic, neurological, or psychiatric diseases. Sixty-one patients (19 females; mean age 72.2 ± 8.8 years) who underwent laminectomy via an interlaminar approach and completed health-related quality of life (HRQoL) assessments both pre- and postoperatively were included in the study. Participants who did not complete the follow-up assessments were excluded from the analyses. Specifically, 17 patients (22% of the total sample) declined to participate in the follow-up and thus were excluded.

The HRQoL and clinical assessment were conducted at two time points: the baseline evaluation was performed within 48 h after surgery and then the follow-up on the thirtieth day after surgery. HRQoL was measured using the Short Form Health Survey 36 (SF-36) which was structured in two macro sections: physical components of SF-36 (PCS) (physical function, role physical, and bodily pain) and mental components of SF-36 (MCS) (general health, vitality, social functioning, role emotional, and mental health) [12]. The clinical evaluations included the Oswestry Disability Index (ODI) and the Roland Morris Disability Questionnaire (RMDQ) to assess disability related to LSS, the Quebec Back Pain Disability Scale (QBPDS) and the Visual Analog Scale (VAS) to measure pain, and the Zung Self-Rating Anxiety Scale (SAS) and Zung Self-Rating Depression Scale (SDS) to assess anxiety and depression, respectively. Demographic and health status information were collected before the LSS intervention (Table 1). The study protocol was approved by the Ethics Committee of University of L’Aquila (protocol n. 07/2022; 1 March 2022).

### Statistical Analysis

The study employed a one-way repeated measure analysis of variance (rmANOVA) to compare the HRQoL and clinical scores before and after surgery. The within-subject variable was the pre–post-surgery evaluation. All values were presented as mean ± SD. Results with *p* < 0.05 were considered statistically significant. Effect sizes, reported as partial eta squared (η^2^p), were classified as small (≥0.01), medium (≥0.06), or large (≥0.14), based on Lakens [13], reflecting the strength of the effect on the dependent variable. Secondly, the changes were quantified using the delta difference (Δ) between the two time points. ΔSF-36 was then compared with Δ of clinical scores and with the baseline demographic variables via Pearson’s correlation analysis to assess the relationship between demographic characteristics, clinical data, and improvements in HRQoL. Correlation strength was classified according to Evans [14]: very weak (<0.20), weak (0.20–0.39), moderate (0.40–0.59), strong (0.60–0.79), and very strong (>0.80). All statistical analyses were conducted using JAMOVI (version 2.3.28.0), an open-source software based on the R framework "(version 2.3.28.0) [15].

## 3. Results

**Figure 1:**
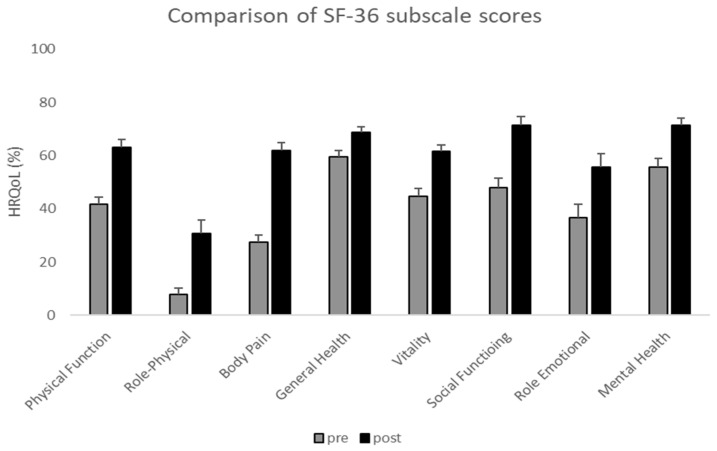
Changes in SF-36 subscale scores before and after LSS surgery. Grey bars represent baseline scores (pre-surgery), and black bars represent scores at 30 days post-surgery. Values are expressed as percentages, and error bars indicate the standard error.

A significant improvement in HRQoL was observed after spinal surgery, with notable gains in both the physical [F(1,60) = 70.3, *p* < 0.001, η^2^p = 0.54] and mental [F(1,60) = 17.9, *p* < 0.001, η^2^p = 0.23] components of the SF-36. All eight SF-36 subscales showed significant improvement (*p* < 0.01), with the largest increase in body pain (+34%), followed by improvements in the domains of physical functioning, role physical, and social functioning (Figure 1). These findings indicate substantial pain relief and improved daily and social functioning after surgery. Similarly, clinical scores demonstrated statistically significant differences (*p* < 0.001) between the preoperative and postoperative assessments following LSS surgery (Table 2).

Significant positive correlations were assessed comparing ΔSF-36 scores of physical and mental health domains with (Δ of clinical scores (Table 3).

Significant correlations were found comparing ΔSF-36 scores of physical and mental health domains with demographic variables and preoperative health status factors. Specifically, a weak negative correlation was found with body mass index (BMI) (r = −0.259, *p* = 0.04), while weak positive correlations were observed with smoking habits (r = 0.288, *p* = 0.02) and psychological distress (r = 0.262, *p* = 0.04). The correlations among the ΔScore of the eight SF-36 subscales are reported in Table 4.

## 4. Discussion

This observational study aimed to assess the impact of preoperative health status and demographic variables on short-term HRQoL in patients with LSS undergoing surgery. Our study demonstrated that after LSS surgery, patients experienced a significant improvement in HRQoL, reflected in both the physical and mental components (PCS and MCS, respectively) of the SF-36, alongside enhancements in clinical scale outcomes, which suggested the efficacy of surgery in improving well-being.

These results were consistent with other previous findings reporting SF-36-related improvements after lumbar surgery [16,17]. However, more recently, it was reported that LSS outcomes after minimally invasive decompression showed post-surgical improvement only in the PCS and not in the MCS ones [18]. Regarding the correlations between improvements in PCS and MCS and clinical outcomes, we observed a significant correlation between enhancements in mental HRQoL and the perception of disability status recorded via the ODI and RMDQ scales. These findings highlighted the need for treatment of emotional, behavioral, and health disorders in patients undergoing LSS surgery, following a growing body of literature emphasizing the requirement of a comprehensive psychological evaluation and management for LSS candidates to surgery [19].

Among the SF-36 subscales, bodily pain showed the most significant improvement, in terms of reduction in its severity, indicating that surgery had a substantial positive effect on pain perception. Improvements in the physical domains of HRQoL were closely associated with a reduction in postoperative pain which undoubtedly played a fundamental role in improving the overall quality of life of patients.

The main finding of our results regarding preoperative health status parameters indicated that only several specific variables determined surgical outcomes related to quality of life. A high preoperative BMI was associated with a lower improvement in the physical component, whereas greater post-surgical benefits were observed in patients without smoking habits or psychological distress, such as depression and anxiety. These results aligned with findings of a recent multicenter study on LSS outcomes following minimally invasive decompression and of data by the Swedish Spine Register, reporting that a higher BMI was linked to poorer clinical outcomes [18,20,21]. When examining the relationships between SF-36 subscales and preoperative health status parameters, it was found that a high BMI had a negative effect on the SF-36′s role physical and role emotional domains. This suggests that people with higher BMIs may have more limitations in their ability to function physically and emotionally. Specifically, they showed more difficulties in performing daily physical activities and may be more likely to experience emotional distress or challenges in social or work roles, affecting their overall quality of life. This could have been due to both the physical strain associated with excess weight and the psychological impact that can accompany a higher BMI.

Regarding psychological distress such as anxiety and depression, our results underline that the presence of these comorbidities negatively affected short-term HRQoL after LSS surgery. Indeed, depression and anxiety were significantly common in patients with low back pain [22,23] and negatively influenced global outcomes in spine surgery [8,24]. In particular, depression and anxiety were associated with a lower quality of life at long-term postoperative follow-up (one year after surgery) compared to patients without these psychological disturbances [25]. Our findings confirmed that psychological distress was also an unfavorable factor in the short-term post-surgical period. Moreover, patients who showed greater improvements in anxiety and depression scores after LSS surgery also exhibited greater gains in HRQoL. A careful diagnosis of psychopathological disorders such as anxiety and depression and their possible therapeutic treatment played a fundamental role in order to avoid the establishment of a vicious circle. In fact, patients with anxiety and depression often had greater difficulty in complying with the protocols required in postoperative recovery and the resulting lack of improvement after surgery may further worsen the anxiety and depressive syndrome maintaining a vicious circle characterized by a heavy health and social burden. Therefore, in the evaluation before surgery for LSS, screening for anxiety-depressive syndrome has necessarily been included.

Smoking status also appeared to influence post-surgical outcomes. Our findings indicated that, although both smokers and non-smokers experienced clinical improvement after LSS surgery, the extent of improvement in HRQoL was slightly reduced among smokers, as also reported in recent clinic research [26]. In fact, smoking contributed to worse post-surgical outcomes, potential complications, increased pain perception, and a poorer subjective response following surgery [27]. Interestingly, tobacco smoking was not only associated with poorer surgical outcomes in terms of HRQoL, as demonstrated by our findings, but also with a higher incidence of surgically treated LSS [28] and an increased likelihood of requiring re-do surgery [29]. These findings underscored the importance of addressing smoking habits as part of the pre- and postoperative management in patients undergoing surgery for LSS.

Sport activity habits were positively correlated with the physical functioning and social role domains of the SF-36, suggesting that individuals who engaged in regular physical activity tended to present better physical functioning and fewer limitations in their social roles. Regular exercise enhanced strength, mobility, and endurance, improving the ability to perform physical tasks and carry out activities of daily living. This highlighted that maintaining an active lifestyle contributed to overall well-being by promoting both physical health and social participation and confirmed that participation in any form of sport or physical activity, whether team or individual, was beneficial for improving mental health and social outcomes among adults [30].

The correlation between the physical functioning domain of the SF-36 and the presence of comorbidities indicated that individuals with these conditions may have experienced greater limitations in physical activities. Comorbidities, such as chronic diseases or other pathologies, could negatively impact overall physical health and functioning, making it more difficult to perform daily tasks. Moreover, our findings indicated that patients with comorbidities experienced a lesser improvement in HRQoL following surgery for LSS than patients without comorbidities. These comorbidities should therefore be considered as important co-factors that influenced surgical outcomes and should be carefully evaluated for their appropriate preoperative resolution or management.

### 4.1. Clinical Implication

The results of this study aim to shift the focus from treating the pathology of LSS to addressing the needs of individuals experiencing specific LSS-related symptoms and disabilities. This aligns with the principles of personalized medicine and the recommendations of the World Health Organization, which emphasize the promotion of patient well-being. Our findings provide meaningful support for the management of surgical LSS patients, particularly in terms of functional outcomes and quality of life. Preoperative consultations that identify such risks can guide a shared decision-making process, enabling both surgeons and patients to make better-informed choices about surgery and its potential outcomes. By recognizing these risk factors in advance, it becomes possible to proactively reduce their impact on quality of life and improve post-surgical outcomes. This approach has recently been tested, showing that prehabilitation interventions (including educational materials, in-person rehabilitation, and telemedicine consultations with a physiatrist) can positively influence the trajectory of care for patients undergoing LSS surgery [31].

### 4.2. Limitations

The improvement in HRQoL was assessed solely in the short term (30 days after surgery). Therefore, any long-term changes in the parameters analyzed were not evaluated. Follow-up evaluations at regular intervals, extending over a longer observation period, could yield valuable insights and contribute to a more comprehensive understanding, ultimately enhancing the surgical outcome. This study identifies associations between variables; however, due to its observational design, it cannot establish causal relationships without longitudinal or interventional evidence. Correlation analysis evaluates the strength and direction of the relationship between two variables. However, the observed dependence is correlational rather than causal, meaning that, while the variables may vary together, one does not necessarily exert a causal influence on the other. It is essential to consider these limitations and potential pitfalls when utilizing and interpreting correlation results [32]. Finally, the relatively small sample size (61 patients) is a general limitation of the study, as it may reduce statistical power and restrict the generalizability of the findings to broader populations.

### 4.3. Future Research Directions

The integration of AI-driven analysis with extensive clinical datasets holds great potential to enhance clinical decision making [33,34], enabling more precise and personalized predictions to support treatment planning and improve HRQoL after LSS surgery. In fact, models incorporating patient demographics, questionnaire data, and MRI findings have shown promising potential for surgical triaging in predicting surgical referrals [35,36]. Comparable models have also been applied to forecast postoperative improvements in HRQoL after surgery in mild degenerative cervical myelopathy [37]. Similar approaches can be implemented in LSS to optimize patient selection and enhance postoperative outcomes by leveraging preoperative status and modifiable factors influencing surgical success.

## 5. Conclusions

A significant improvement in HRQoL and general clinical status was recorded after LSS surgery, particularly with respect to severity of pain control. However, several preoperative health status factors could impact the quality-of-life outcome after LSS surgery. Psychological distress, BMI, tobacco smoke, and comorbidity can negatively influence surgical outcomes, in both physical and mental components of HRQoL. On the contrary, sports routines seem to increase the HRQoL after LSS, in relation to all-day life and work activities and in the social interactions. The current findings underscore the importance of a comprehensive preoperative assessment including habits, lifestyle, and comorbidities in patients undergoing surgery for LSS. A comprehensive assessment helps identify individuals who are most likely to benefit from intervention, improving prognostic accuracy and supporting clinical decision making to optimize HRQoL outcomes.

## Figures and Tables

**Table 1:** Sample characteristics.

**Demographic** **variables**	Age (years)	72.21 ± 8.8
	Sex (F; M)	19 (31.1%); 42 (68.9%)
	Weight (Kg)	81.18 ± 14.8
	Height (cm)	170.16 ± 10.6
	BMI	27.45 ± 4
	Living alone (no; yes)	49 (80.3%); 12 (19.7%)
	Instruction level (≤5 years; >5–≤8 years; >8–≤13 years; >13 years)	11 (18.0%); 20 (32.8%); 21 (34.4%); 9 (14.7%)
**Health status** **information**	Physically demanding work (no; yes)	26 (42.6%); 35 (57.4%)
	Smoking habits (no; ex-smokers; yes)	18 (29.5%); 11 (18.0%); 32 (52.5%)
	Pain onset (months)	56.66 ± 110.4
	Complex drug therapy (no; yes)	36 (59%); 25 (41.0%)
	Sport activity (no; yes)	31 (50.8%); 30 (49.2%)
	History of back pain (no; yes)	11 (18.0%); 50 (82.0%)
	Physiological distress (no; yes)	42 (68.8%); 19 (31.2%)
	Diabetes (no; yes)	49 (80.3%); 12 (19.7%)
	Rheumatic disease (no; yes)	59 (96.7%); 2 (2.3%)
	Comorbidity (more than 4) (no; yes)	41 (67.2%); 20 (32.8%)

Abbreviations: BMI: body mass index.

**Table 2:** Change in clinical scores before and after LSS surgery.

	ODI Pre	ODI Post	RMDQ Pre	RMDQ Post	QBPDS Pre	QBPDS Post	VAS Pre	VAS Post	SAS Pre	SAS Post	SDS Pre	SDS Post
**Mean ± DS**	45.1 ± 18.8	24.2 ± 17.8	15.8 ± 4.86	10.6 ± 5.13	45.2 ± 18.2	28.5 ± 18.1	6.62 ± 2.58	3.05 ± 2.49	36.8 ± 8.5	30.7 ± 7.65	37.8 ± 10.5	32.8 ± 9.31
**Minimal value**	0	0	4	1	1	1	0	0	21	21	22	20
**Maximal value**	88	76	23	22	96	78	10	10	62	56	66	57
**Shapiro–Wilk W**	0.989	0.95	0.959	0.975	0.987	0.943	0.916	0.919	0.97	0.892	0.957	0.938
**Shapiro–Wilk p**	0.867	0.015	0.039	0.242	0.746	0.007	<0.001	< 0.001	0.136	<0.001	0.033	0.004
***p* value**		<0.001		<0.001		<0.001		<0.001		<0.001		<0.001
**η^2^p**		0.56		0.49		0.46		0.6		0.34		0.27

Abbreviations: ODI: Oswestry Disability Index; RMDQ: Roland Morris Disability Questionnaire; QBPDS: Quebec Back Pain Disability Scale; VAS: Visual Analog Scale; SAS: Zung Self-Rating Anxiety Scale; SDS: Zung Self-Rating Depression Scale.

**Table 3:** Correlations between SF-36 physical and mental domains and clinical scores.

ΔScore SF-36 Components	ΔScore Clinical Scales	r	*p*	Interpretation
PCS	ODI	0.354	0.005	Weak
MCS	ODI	0.535	<0.001	Moderate
PCS	RMDQ	0.298	0.02	Weak
MCS	RMDQ	0.474	<0.001	Moderate
PCS	QBPDS	0.256	0.04	Weak
MCS	QBPDS	0.499	<0.001	Moderate
PCS	VAS	0.415	<0.001	Moderate
MCS	VAS	0.293	0.02	Weak
PCS	SAS	0.546	<0.001	Moderate
MCS	SDS	0.402	0.001	Moderate

Abbreviations: PCS: physical components of SF-36; MSC: mental components of SF-36; ODI: Oswestry Disability Index; RMDQ: Roland Morris Disability Questionnaire; QBPDS: Quebec Back Pain Disability Scale; VAS: Visual Analog Scale; SAS: Zung Self-Rating Anxiety Scale; SDS: Zung Self-Rating Depression Scale. The variable r denotes the correlation coefficient, while p indicates the associated statistical significance.

**Table 4:** Correlations between SF-36 physical and mental domains and preoperative health status.

ΔScore SF-36 Subscales	Preoperative Variable	r	*p*
Physical function	Sport activity	0.38	0.003
Physical function	Comorbidity	−0.259	0.04
Role physical	BMI	−0.368	0.004
Role physical	Weight	−0.283	0.03
Social function	Sport activity	0.331	0.009
Role emotional	BMI	−0.302	0.02
Role emotional	Weight	−0.286	0.03

Abbreviations: BMI: body mass index. The variable r denotes the correlation coefficient, while *p* indicates the associated statistical significance.

## Data Availability

The raw data supporting the conclusions of this article will be made available by the authors on request. The data are not publicly available due to local privacy policy.

## Abbreviations

The following abbreviations are used in this manuscript:

HRQoL	Health-related quality of life
LSS	Lumbar spinal stenosis
SF-36	Short Form Health Survey 36
PCS	Physical components of SF-36
MCS	Mental components of SF-36
ODI	Oswestry Disability Index
RMDQ	Roland Morris Disability Questionnaire
QBPDS	Quebec Back Pain Disability Scale
VAS	Visual Analog Scale
SAS	Zung Self-Rating Anxiety Scale
SDS	Zung Self-Rating Depression Scale
BMI	Body mass index
